# Factors Determining Rehabilitation Needs After Intradural Spinal Tumor Surgery: A Prospective Study

**DOI:** 10.3390/brainsci15010051

**Published:** 2025-01-08

**Authors:** Stanisław Krajewski, Jacek Furtak, Monika Zawadka-Kunikowska, Michał Kachelski, Jakub Soboń, Marek Harat

**Affiliations:** 1 Centre of Medical Sciences, Jan and Jędrzej Śniadeccy University of Science and Technology, 85-796 Bydgoszcz, Poland; jacek.furtak2019@gmail.com (J.F.); marek.harat@10wsk.mil.pl (M.H.); 2 Department of Neurosurgery, 10th Military Research Hospital and Polyclinic, 85-681 Bydgoszcz, Poland; kachelskim@gmail.com (M.K.); drsobon@gmail.com (J.S.); 3 Department of Human Physiology, Ludwik Rydygier Collegium Medicum in Bydgoszcz Nicolaus Copernicus University in Torun, 85-092 Bydgoszcz, Poland; m.zkunikowska@cm.umk.pl

**Keywords:** intradural tumor surgery, functional state, histopathological subtype, rehabilitation

## Abstract

Background/Objectives: While most studies on the postoperative condition of patients with spinal cord tumors describe long-term outcomes, data are needed on immediate surgical outcomes demanding rehabilitation to make informed assessments for postoperative planning. The aim of this study was to identify factors predicting function and rehabilitative needs after intradural spinal tumor surgery. Methods: Eighty-five prospectively recruited patients underwent surgery for intradural intramedullary (ID-IM; *n* = 23) and extramedullary (ID-EM; *n* = 62) tumors. Neurological and functional status were assessed before surgery, after surgery, and at discharge using the modified McCormick scale (MMS), Karnofsky performance status (KPS) scale, Barthel index (BI), and the gait index (GI). Results: There were no significant predictors of early postoperative rehabilitation in the ID-IM group. In the ID-EM group, age, thoracic level, subtotal resection (STR), repeat surgery, and functional scale scores predicted the need for rehabilitation. In multivariable analysis, MMS (odds ratio (OR) 8.7; 95% confidence interval (CI): 2.37–32.44) and STR (OR 13.00; 95%CI: 1.56–107.87) remained independent predictors of rehabilitation need (area under curve, 92%). Despite their younger age, most patients with ID-IM tumors, especially ependymomas, required rehabilitation but improved quickly (KPS, BI, *p* < 0.001). Among ID-EM tumors, meningiomas were characterized by poorer preoperative function and low gross total resection (GTR) rates, but did not deteriorate neurologically after surgery. Patients with schwannoma and ID-EM ependymomas achieved the highest GTR rate and had the best function both before and after surgery. Conclusions: These results may be useful for estimating early rehabilitation needs after intradural tumor surgery and counseling patients before surgery about the expected postoperative course.

## 1. Introduction

Tumors of the central nervous system (CNS) constitute only 1.5–2% of all cancers [1,2,3], with primary spinal cord tumors representing just 2–4% of CNS tumors [4,5,6,7]. As they are so rare, population-based data on these tumors are limited [8]. Patient outcomes depend on factors such as tumor location (intramedullary or extramedullary), histopathology, growth rate, spinal level [4,9,10,11], the patient’s age and general condition prior to surgery [10,11,12], and access to advanced diagnostic and surgical equipment, including intraoperative monitoring [13,14]. Surgery is typically the treatment of choice for spinal tumors [4,15], with 59–96% of patients experiencing a positive surgical outcome, such as gross total resection (GTR) [10,13,14,15,16,17]. In over 90% of cases, function remains stable or improves, although some patients require postoperative rehabilitation [5,7,9,18,19]. This rehabilitation is generally effective, allowing many patients to return to professional and even sporting activities [20,21,22].

Intradural tumors are classified based on their location relative to the spinal cord: intramedullary (ID-IM) or extramedullary (ID-EM). Ependymomas, which are usually non-malignant [8,17,23,24,25], typically present around age 45, more often in men, and are commonly located in the cervical spine. When surgery is performed before neurological deficits develop, postoperative function is generally favorable [17,19]. Schwannomas and meningiomas are the most common ID-EM tumors, with schwannomas showing low rates of motor deficit incurrence, high GTR success (>90%) [4,26,27], and sporadic recurrences [6,13]. Although benign, meningiomas (>80% thoracic, more common in females (over 80%) [5,28]) can be challenging to manage due to their slow growth and consequent progressive neurological deficits before surgery [11,12,29,30,31,32].

Most studies on spinal tumors focus on long-term postoperative outcomes, with patients often showing significant improvements months or years after surgery. However, there is a lack of data on the condition of patients immediately after surgery, which serves as the starting point for postoperative rehabilitation and is essential for informed postoperative planning. The main objective of this study was to identify predictors of the need for postoperative rehabilitation in patients undergoing surgery for intradural spinal tumors. The secondary goal was the assessment of the relationships between tumor type and neurological status, functional performance, activities of daily living (ADL), and gait efficiency preoperatively and in the early postoperative period. These results may be useful for estimating early rehabilitation needs and counseling patients before surgery about the expected postoperative course.

## 2. Materials and Methods

### 2.1. Patients

**Figure 1:**
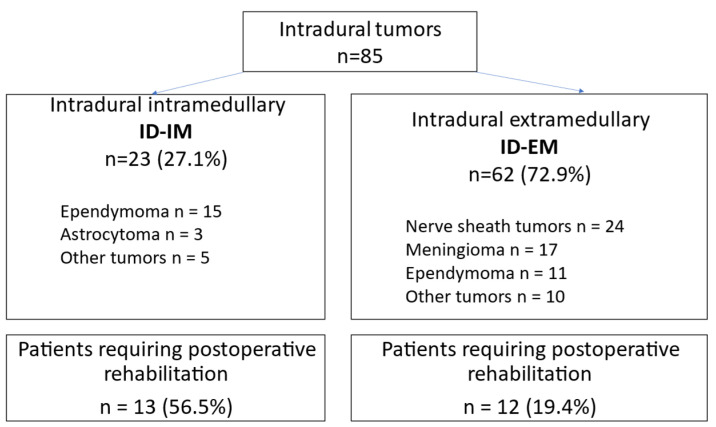
Flow chart of study participants.

The Bioethics Committee at the Military Medical Chamber approved the study protocol (no. 164/18). All patients provided informed consent. This was a single-center, prospective, observational controlled study with follow-up time from the day of admission to hospital to the day of discharge (3–21 days). Patients undergoing surgery for intradural tumors (*n* = 85) from August 2018 to November 2021, including 23 (27.1%) patients with ID-IM tumors and 62 (72.9%) patients with ID-EM tumors, were evaluated. Twenty-five patients (29.4%) required rehabilitation during their inpatient stay at the Neurosurgery Clinic (Figure 1) and included patients with preoperative neurological deficits and those who developed new deficits after surgery, most commonly paresis and paralysis of the limbs, deep sensory disturbances resulting in ataxia, and impaired motor coordination. Rehabilitation began on the first day after surgery at the request of the attending physician. Patients were rehabilitated using the proprioceptive neuromuscular facilitation (PNF) method, which is especially indicated for paresis, paralysis, and deep sensory disorders. The PNF method involves performing a strict set of exercises that replicate natural movements [33]. Patients who did not require specialized rehabilitation received postoperative support during the first upright positions and attempts to walk.

The tumor subtype and WHO grade were established by histopathological examination. All surgeries were performed under general anesthesia. The level of surgery was determined in the operating room by fluoroscopy. Intramedullary tumors were removed by central myelotomy. During all surgeries, intraoperative potentials were used (somatosensory evoked potentials, motor evoked potentials, and T-wave, according to individual need).

The inclusion criteria for the ID-IM and ID-EM groups were as follows: primary intradural intramedullary or intradural extramedullary tumor surgery, respectively; initial or repeat surgery; age of at least 18 years old; and informed consent having been provided to participate in the study.

### 2.2. Patient Assessment

Neurological and functional status were assessed with the modified McCormick scale (MMS); general condition and performance in cancer with the Karnofsky Performance Status (KPS) scale; ADL with the Barthel index (BI); gait efficiency with the gait index (GI) [15,34,35,36]; and muscle strength with the Lovett scale (a scale with 1-point increments, where 0 is paralysis and 5 is full strength). Assessments were performed three times: preoperatively, immediately after surgery, and at discharge. Data regarding the level of surgery, the number of levels of extension-requiring surgery, and the extent of tumor resection (GTR or subtotal resection (STR)) were obtained from the operative notes. The extent of resection was confirmed by contrast-enhanced MRI on the first postoperative day.

### 2.3. Outcomes

The primary outcome was participation in postoperative rehabilitation, and secondary outcomes were neurological and functional status before and after tumor resection. As ID-IM and ID-EM tumors are fundamentally different in terms of presentation, anatomy, surgical requirements, and histological/biological spectrum, we considered these as two different subgroups.

### 2.4. Statistical Analysis

All data were analyzed using the PS IMAGO v10.0 and Statistica 13.0 PL statistical packages. Descriptive statistics summarizing patient demographics are presented as mean ± SD or number (%). The Shapiro–Wilk test was used to verify the normality of the distribution of variables, and as the data were not normally distributed, differences between two continuous variables were assessed using the Mann–Whitney U test. Chi-squared or Fisher’s exact tests were used to determine relationships between categorical variables. Associations between selected variables (age, spine level, preoperative MMS, KPS, BI, GI scores, extent of tumor resection, type of surgery (initial/repeat), and histopathology) and the need for postoperative rehabilitation were evaluated separately for the ID-IM and ID-EM groups using univariable logistic regression. Multivariable logistic regression models were constructed by selecting variables using a stepwise forward selection method. Receiver operating characteristic (ROC) curve analysis and area under the curve (AUC) calculations were performed for the constructed models, with AUC values compared using the Z-test. The non-parametric Friedman ANOVA for repeated measures was applied to evaluate the time effect (preoperatively/after surgery/at discharge) on functional activity (MMS, BI, KPS, and GI) with Bonferroni correction and Kruskal–Wallis tests for group effects. A *p*-value < 0.05 was considered significant.

## 3. Results

### 3.1. Clinical and Demographic Characteristics of ID-IM and ID-EM Subgroups

Twenty-three patients with ID-IM tumors and 62 with ID-EM tumors were recruited to the study (Figure 1).

In the ID-IM group, the mean patient age was 43.3 years, and the majority of lesions were at the cervical spine level (69.6%). Surgery involved one spinal level in 47.8% of cases and more than one level in 52.2% of cases. The ID-IM group was characterized by a high percentage of GTR (78.3%) and a low frequency of repeat surgery (17.4%). Muscle weakness (<5 in Lovett scale) was present in 17.4% of patients prior to surgery, and 73.9% of patients improved or remained unchanged at discharge. Neurological status, as assessed by the MMS, was 1.6 before surgery and 1.9 at discharge (*p* > 0.05). Thirteen patients required early postoperative rehabilitation.

Of the ID-IMs, 15 (65.2%) were ependymomas (14 WHO grade II and 1 anaplastic WHO grade III) and 8 were other tumor subtypes. Patients with ependymoma had generally good surgical outcomes: 80% GTR and 6.7% repeat surgery, and ten patients (66.7%) required rehabilitation (Appendix A, Table A1).

In the ID-EM group, there were 24 (38.7%) nerve sheath tumors (NSTs, WHO grade I), 17 (27.4%) meningiomas (WHO grade I except for one atypical WHO grade II tumor), 11 (17.7%) ependymomas (WHO grade II), and 10 (16.1%) tumors belonging to other subtypes. Surgery involved one spinal level in 40.3% of cases and more than one level in 59.8% of cases. Meningiomas were significantly more common in females than males and tended to occur in older individuals. There were no statistically significant differences between tumor subtype with respect to extent of tumor resection, repeat surgery, spinal level (cervical/cervicothoracic), muscle strength (Lovett scale), MMS, or need for rehabilitation, but meningiomas were more common in the thoracic/thoracolumbar region and ependymomas in the lumbar/lumbosacral region (*p* = 0.027 and *p* = 0.001, respectively). Motor weakness was observed in 33.9% of patients before surgery, and this improved or remained unchanged at discharge in 95.2% of cases. The mean MMS neurological status was 1.6 before surgery and 1.5 at discharge (*p* > 0.05). Twelve ID-EM patients required rehabilitation after surgery (Appendix B, Table A2).

### 3.2. Comparison of Clinical Factors Determining the Need for Postoperative Rehabilitation After Tumor Surgery

The degree of resection, initial/repeat surgery, and spinal level were associated with the need for postoperative rehabilitation for ID-EM tumors. Preoperative neurological status (MMS), performance (KPS), ADLs (BI), and gait (GI) were also significantly different (*p* < 0.001) between ID-EM patients requiring and not requiring postoperative rehabilitation. Patients with ID-EM tumors requiring rehabilitation were significantly older than those not requiring rehabilitation (*p* = 0.024). However, there were no such relationships for patients with ID-IM tumors (Table 1).

### 3.3. Prediction of Need for Postoperative Rehabilitation

For patients with ID-IM tumors, there were no significant predictors of early postoperative rehabilitation. For patients with ID-EM tumors, there were significant differences for all variables except for the histopathological diagnosis. The odds of requiring postoperative rehabilitation increased by 5.3% for each additional year of age (OR = 1.05), and the odds of requiring rehabilitation were significantly higher in patients who underwent thoracic spine surgery than those who underwent cervical or lumbar spine surgery (OR = 23.4). STR carried 6.1-times greater odds of postoperative rehabilitation than GTR (OR = 6.1), and patients undergoing repeat surgery had 4.7-times greater odds than those undergoing initial surgery (OR = 4.7). Additionally, for each unit increase in the MMS score, the odds of needing postoperative rehabilitation increased nearly seven-fold (OR = 7.0) (Table 2). The risks associated with KPS, BI, and GI were similar and significant.

Subsequently, multivariable logistic regression models were constructed by selecting variables using a stepwise forward selection method. Independent models were constructed for the ID-IM and ID-EM groups. For the ID-EM dataset, the final model included two variables: MMS and extent of resection. Statistically significant regression parameter estimates were observed for the ID-EM model while, for the ID-IM model, they were not significant.

In the ID-EM model, the need for early postoperative rehabilitation increased 8.8-fold with each unit increase in MMS, adjusting for the extent of resection. The need for rehabilitation in the STR group was over 13-times greater than in the GTR group, adjusting for MMS (Table 3).

### 3.4. ROC Curve Analysis

The ID-EM model showed a high predictive power (AUC = 0.929), while the ID-IM model showed only limited predictive power (AUC = 0.619). There were significant differences in AUCs, indicating that the ID-EM model was more accurate (Z-test = −2.51, *p* = 0.012); AUC 92%).

### 3.5. Functional State Before Surgery, After Surgery, and at Discharge for Patients Undergoing Surgery for Common Histopathological Subtypes of Tumors

**Figure 2:**
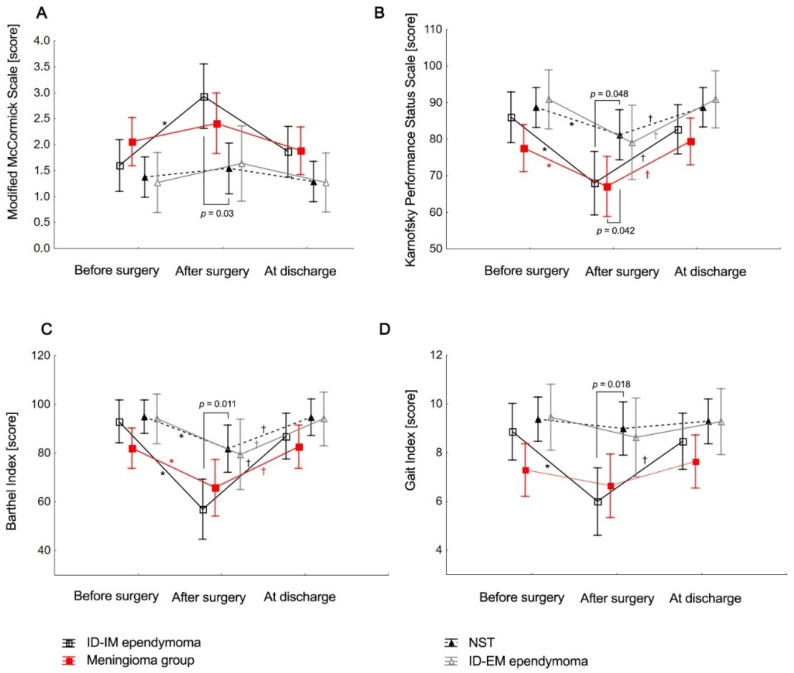
(**A**) Neurological status (MMS), (**B**) performance (KPS), (**C**) activities of daily living (BI), and (**D**) gait efficiency (GI) before surgery, after surgery, and at discharge.

The functional status of patients with the four most common histopathological subtypes of tumors (ID-IM ependymoma, meningioma, NST, and ID-EM ependymoma) was compared before surgery, immediately after surgery, and at discharge. There were significant differences between the groups after surgery. NST patients showed better functional outcomes than ID-IM ependymoma patients across all scales (KPS, BI, GI, MMS) and compared with the meningioma group for the KPS scale. The ID-IM ependymoma group experienced overall post-surgery deterioration across scales (before vs. after MMS: *p* = 0.006; KPS: *p* = 0.001; BI: *p* = 0.001; GI: *p* = 0.002) but showed better KPS and BI outcomes at discharge compared with after surgery (*p* < 0.001). Both the meningioma and NST groups showed deterioration in KPS and BI after surgery compared with before surgery (*p* = 0.014; *p* = 0.024) and improvements at discharge compared with after surgery (*p* = 0.002; *p* = 0.008). The ID-EM ependymoma group had better KPS (*p* = 0.017) and BI (*p* = 0.032) scores at discharge compared with after surgery (Table 4, Figure 2).

Finally, we confirmed that the need for rehabilitation significantly extended the length of the hospital stay. In the ID-IM group, the mean inpatient stay of those who required rehabilitation was 9.4 ± 3.50 days vs. 6.3 ± 1.83 days for those who did not need rehabilitation (*p* = 0.019). In the ID-EM group, these times were 9.9 ± 4.52 and 6.2 ± 1.93 days, respectively (*p* < 0.001).

## 4. Discussion

Here, we evaluated the pre- and postoperative function of patients undergoing intradural tumor surgery. As postoperative deterioration poses a significant clinical and rehabilitative challenge, our goal was to identify predictors of postoperative rehabilitation needs based on tumor type, location, resection extent, surgery type, and patient condition. We found no significant predictors of need for early postoperative rehabilitation in the ID-IM group, as surgery for these tumors was such a significant factor influencing postoperative functional status. In the ID-EM group, age, thoracic level, STR, repeat surgery, and functional scale scores predicted the need for rehabilitation in univariable analysis. In multivariable analysis, MMS and STR remained independent predictors.

The characteristics of our patients with ependymoma were similar to those in published data [5,6,15] (65.2% of operated ID-IM tumors, average age 44 years). Neurological condition, performance, ADLs, and gait were slightly impaired before the procedure, which significantly determined favorable function after surgery [14,17,19]. Ependymomas were most commonly sited in the cervical and cervicothoracic sections (80%), contributing to a high proportion of GTRs (80%) and a low proportion of repeat operations (6.7%) [9], similar to data from other centers [10,15,16,17,24]. However, despite these favorable characteristics, two-thirds of patients required rehabilitation after ependymoma surgery. Brotchi et al. reported that most patients with ependymomas experience functional deterioration immediately after surgery because of separation of the posterior columns, resulting in deep sensory deficits. In most cases, recovery was observed within one to three months [14,17]. Here, immediately after surgery, neurological and functional metrics deteriorated more for patients in the ependymoma group than for the other ID-EM tumor subtypes, but by discharge they had returned to admission levels. Of note, intramedullary intervention resulted in a deterioration in function regardless of whether GTR was achieved, it was the first or repeat surgery, or the affected spinal level.

For ID-EM tumors, several factors significantly increased the need for postoperative rehabilitation: (i) in patients with STR, the odds of requiring rehabilitation were 6.1-times higher than in patients achieving GTR (OR = 6.1); (ii) in patients undergoing repeat surgery, the odds of requiring rehabilitation were significantly higher than in those undergoing first surgery (OR = 4.7); (iii) almost all rehabilitated patients had tumors in the thoracic spine, with only one having a tumor in the lumbar spine and none in the cervical spine (OR = 23.4); (iv) patients who underwent rehabilitation were older (62.2 years vs. 50.6 years) than those who did not, and requirements for rehabilitation increased by 5.3% for each additional year of age; and (v) rehabilitated patients were in worse neurological and functional condition according to all scales prior to surgery than those who did not require rehabilitation (*p* < 0.001), especially for the MMS (OR = 7.0).

Although these unfavorable factors were most common in patients with meningioma, the low GTR rate (58.8%) may be surprising. This may be attributed to the high repeat surgery rate (35.3%) and frequent thoracic spine location (over 70%) in this group, which are poor prognostic factors [9,37,38]. A full appraisal of surgical efficacy, however, would require evaluating two additional factors unavailable in our study: tumor volume and the precise location of the tumor relative to the spinal cord. Moreover, patients with meningioma most often had preoperative motor deficits (41.2%), and the neurological status was worse (MMS = 2.1) than the other subtypes, probably due to their slow growth leading to only gradual, imperceptible progression of neurological symptoms but deteriorated function [29]; indeed, Haegelen et al. reported 57% paraparesis and 37% paraplegia, Narayan et al. reported 80% motor deficits, and Solero et al. reported 30% significant to severe neurological impairment up to paraplegia in patients scheduled for surgery for meningioma [11,30,39]. Older age (average 64 years) was another unfavorable factor [12]. Despite these challenges, 17.6% of patients improved, and 82.4% maintained preoperative function. Our data support that meningioma surgery is safe and yields good outcomes in older patients [32,38].

Patients with schwannoma and other NSTs had distinct outcomes. Schwannoma cases showed the highest GTR rate (95.2%), consistent with Ottenhausen et al. (96%) and Hohenberger et al. (90%) [6,13]. Recurrences were rare, and good neurological status pre- and post-surgery minimized rehabilitation needs. Similarly, 81.8% of lumbar ID-EM ependymomas achieved GTR, with most patients showing improvement or stability.

Postoperative inpatient rehabilitation should be comprehensive and include all patients with neurological deficits [7,40,41]; however, prognostic factors should be taken into account in the planning and distribution of rehabilitation services [42]. A significant percentage of patients with spinal diseases and injuries qualifying for rehabilitation have intradural tumors [43], but our work shows that this group is very heterogeneous in terms rehabilitative needs.

Our research has several limitations. The first is the short follow-up, due to the nature of this observational, prospective study. Few neurosurgical centers perform so many surgeries on these rare tumors that it is possible to collect sufficient data for prospective statistical analysis, but nevertheless prospective studies are needed to reduce bias. Second, the small and unequal size of the groups meant that despite the clearly observed differences, the analysis may be underpowered and there may be some bias. Third, we did not consider the surgical approach and other technical aspects of surgery that determine postoperative outcomes, since this was not directly relevant to rehabilitative needs. Fourth, we did not consider the effects of adjuvant treatment, as this is not directly relevant to predictors of early postoperative rehabilitation. Finally, while we considered the intra- or extramedullary location, the relationship of the tumor to the spinal cord (anterior, posterior, lateral) and the tumor volume were not considered, which is important in terms of GTR, as these data were not available.

## 5. Conclusions

There were no significant predictors of early postoperative rehabilitation in ID-IM tumor patients. In the ID-EM group, factors such as age, thoracic level, STR, repeat surgery, and preoperative functional scores predicted rehabilitation needs. Multivariable analysis identified MMS and STR as independent predictors for ID-EM patients. ID-IM tumors, particularly ependymomas, posed greater postoperative functional challenges despite a higher likelihood of GTR, with most patients requiring rehabilitation but showing rapid improvement. In contrast, meningioma patients—who most often required rehabilitation among patients with ID-EM tumors—had the worst preoperative function and a lower likelihood of achieving GTR; however, their neurological status did not deteriorate after surgery. Patients with schwannomas and ID-EM ependymomas had the best surgical outcomes. These findings help estimate rehabilitation needs and guide preoperative counseling, though larger confirmatory studies are needed.

## Figures and Tables

**Table 1:** Factors associated with postoperative rehabilitation after ID-IM and ID-EM tumor surgery.

Characteristic	ID-IM			ID-EM		
	Need for Rehabilitation	No Need for Rehabilitation	*p*-Value	Need for Rehabilitation	No Need for Rehabilitation	*p*-Value
*n* (%)	13 (56.5)	10 (43.5)		12 (19.4)	50 (80.6)	
Gender						
Female *n* (%)	6 (60.0)	4 (40.0)	0.768	8 (21.6)	29 (78.4)	0.582
Male *n* (%)	7 (53.8)	6 (46.2)		4 (16.0)	21 (84.0)	
Spinal Level						
C + CTh *n* (%)	9 (56.3)	7 (43.7)		0 (0)	11 (100)	
Th + ThL *n* (%)	4 (57.1)	3 (42.9)	0.968	11 (40.7)	16 (59.3)	0.001
L+ LS *n* (%)	0 (0)	0 (0)		1 (4.2)	23 (95.8)	
Range of Resection						
GTR *n* (%)	10 (55.5)	8 (44.5)	0.859	6 (12.2)	43 (87.8)	0.006
STR *n* (%)	3 (60.0)	2 (40.0)		6 (46.2)	7 (53.8)	
Initial/Repeat Surgery						
Initial *n* (%)	11 (57.9)	8 (42.1)	0.772	4 (10.3)	35 (89.7)	0.018
Repeat *n* (%)	2 (50.0)	2 (50.0)		8 (34.8)	15 (65.2)	
Histopathology						
Meningioma *n* (%)	0 (0)	0 (0)		6 (35.3)	11 (64.7)	
NST *n* (%)	0 (0)	0 (0)	0.178	2 (8.3)	22 (91.7)	0.199
Ependymoma *n* (%)	10 (66.7)	5 (33.3)		2 (18.2)	9 (81.8)	
Other *n* (%)	3 (37.5)	5 (62.5)		2 (20.0)	8 (80.0)	
State Before Surgery						
Age, Mean ± SD	44.6 ± 14.45 **	41.5 ± 9.45	0.563	62.2 ± 14.56 **	50.6 ± 15.77	0.024
MMS, Mean ± SD	1.7 ± 1.07 *	1.2 ± 0.40	0.177	2.9 ± 1.26 *	1.3 ± 0.54	<0.001
KPS, Mean ± SD	84.6 ± 13.37 ^†^	92.0 ± 7.48	0.132	66.7 ± 20.1 ^†^	88.2 ± 8.64	<0.001
BI, Mean ± SD	91.9 ± 17.27 ^^^	99.0 ± 3.00	0.215	73.3 ± 24.86 ^^^	92.8 ± 12.17	<0.001
GI, Mean ± SD	8.8 ± 2.12 ^×^	9.7 ± 0.46	0.203	5.8 ± 3.17 ^×^	9.3 ± 1.37	<0.001

Abbreviations: NST, nerve sheath tumor; GTR, gross total resection; STR, subtotal resection; C, cervical; Th, thoracic; L, lumbar; S, sacral; MMS, modified McCormick scale; KPS, Karnofsky performance status; BI, Barthel index; GI, gait index. Notes: differences between rehabilitated ID-IM and ID-EM patients: * MMS, *p* = 0.017; ^†^ KPS, *p* = 0.015; ^^^ BI, *p* = 0.039; ^×^ GI, *p* = 0.010; ** Age, *p* = 0.006.

**Table 2:** Univariable logistic regression analysis: need for postoperative rehabilitation in patients with ID-IM and ID-EM tumors.

	ID-IM				ID-EM			
	OR	Lower 95% CI	Upper 95% CI	*p*-Value	OR	Lower 95% CI	Upper 95% CI	*p*-Value
Age	1.02	0.96	1.09	0.559	1.05	1.00	1.11	0.035
Level Th	1.04	0.17	6.23	0.968	23.38	2.77	197.02	0.004
Extent-STR	1.20	0.16	9.01	0.859	6.14	1.54	24.54	0.010
Repeat Surgery	2.70	0.24	30.85	0.424	4.67	1.22	17.90	0.025
MMS	2.38	0.57	9.94	0.234	6.98	2.31	21.10	0.001
KPS	0.93	0.84	1.03	0.168	0.90	0.84	0.96	0.001
BI	0.93	0.79	1.08	0.337	0.95	0.91	0.98	0.003
GI	0.55	0.17	1.74	0.309	0.52	0.35	0.78	0.001
Histopathology	0.30	0.05	1.80	0.187	0.82	0.10	7.02	0.855

Abbreviations: OR, odds ratio; CI, confidence interval; STR, subtotal resection; MMS, modified McCormick scale; KPS, Karnofsky performance status; BI, Barthel index; GI, gait index.

**Table 3:** Multivariable logistic regression analysis: need for postoperative rehabilitation in ID-IM and ID-EM patients.

Predictor	Multivariable Logistic Regression Model							
	Level	B	SE	Wald	*p*-Value	OR	Lower 95% CI	Upper 95% CI
ID-EM								
MMS		2.17	0.67	10.58	0.001	8.77	2.37	32.45
Extent of Resection	STR	2.57	1.08	5.65	0.017	13.00	1.57	107.88
ID-IM								
MMS		−0.82	1.31	0.39	0.533	0.44	0.03	5.78
Extent of Resection	STR	1.05	0.77	1.85	0.174	2.85	0.63	12.92

Abbreviations: B: unstandardized regression coefficient; SE, standard error; OR, odds ratio; 95% CI, confidence interval.

**Table 4:** Neurological status (MMS), performance (KPS), activities of daily living (BI), and gait efficiency (GI) before surgery, after surgery, and at discharge according to major histopathological subtype.

Scale	Time	ID-IM Ependymoma *n* = 15	Meningioma *n* = 17	NST *n* = 24	ID-EM Ependymoma *n* = 11
		Mean ± SD	Mean ± SD	Mean ± SD	Mean ± SD
MMS	Before Surgery	1.6 ± 1.02 *	2.1 ± 1.26	1.4 ± 0.70	1.3 ± 0.62
	After Surgery	2.9 ± 1039 *	2.4 ± 1.29	1.5 ± 0.91	1.6 ± 1.15
	At Discharge	1.9 ± 0.96	1.9 ± 1.13	1.3 ± 0.84	1.3 ± 0.62
*p*-Value		<0.001	0.004	0.029	0.148
KPS	Before Surgery	86.0 ± 13.06	77.6 ± 18.32 *	88.8 ± 9.27 *	90.9 ± 9.00
	After Surgery	68.0 ± 13.27	67.1 ± 17.74 *^,†^	81.3 ± 16.15 *^,†^	79.1 ± 18.32 ^†^
	At Discharge	82.7 ± 13.89	79.4 ± 14.74 ^†^	88.8 ± 10.53 ^†^	90.9 ± 11.64 ^†^
*p*-Value		<0.001	<0.001	<0.001	0.002
BI	Before Surgery	93.0 ± 16.31	82.1 ± 24.56 *	95.0 ± 9.57 *	94.1 ± 12.58
	After Surgery	57.0 ± 23.37	65.9 ± 24.81 *^,†^	81.9 ± 21.59 *^,†^	79.5 ± 23.78 ^†^
	At Discharge	87.0 ± 21.20	82.6 ± 23.33 ^†^	94.8 ± 12.37 ^†^	94.1 ± 11.04 ^†^
*p*-Value		<0.001	<0.001	<0.001	0.001
GI	Before Surgery	8.9 ± 2.00 *	7.3 ± 3.48	9.4 ± 1.18	9.5 ± 1.23
	After Surgery	6.0 ± 2.66 *	6.7 ± 3.31	9.0 ± 1.96	8.6 ± 2.50
	At Discharge	8.5 ± 1.82	7.7 ± 3.12	9.3 ± 1.84	9.3 ± 1.36
*p*-Value		<0.001	0.016	0.074	0.148

Abbreviations: MMS, modified McCormick scale; KPS, Karnofsky performance status; BI, Barthel index; GI, gait index. * indicates before vs. after *p* < 0.05. ^†^ indicates after vs. at discharge *p* < 0.05.

## Data Availability

All the data are presented within the manuscript.

## Appendix A

**Table A1:** Characteristics of the ID-IM group.

	Ependymoma	Other Tumors *	Total
*n*, (%)	15 (65.2)	8 (34.8)	23 (100)
Female, *n* (%)	6 (40.0)	4 (50.0)	10 (43.5)
Male, *n* (%)	9 (60.0)	4 (50.0)	13 (56.5)
Age, Mean ± SD [Range]	44.3 ± 11.73 [24–61]	41.4 ± 13.94 [19–57]	43.3 ± 12.62 [19–61]
MMS Before Surgery, Mean ± SD	1.6 ± 1.01	1.3 ±0.43	1.5 ± 0.88
MMS at Discharge, Mean ± SD	1.9 ± 0.96	1.6 ± 0.70	1.8 ± 0.88
Length of Hospital Stay, Mean ± SD	8.4 ± 3.24 [5–17]	7.4 ± 3.62 [4–15]	8.0 ± 3.24 [4–17]
GTR, *n* (%)	12 (80.0)	6 (75.0)	18 (78.3)
STR, *n* (%)	3 (20.0)	2 (25.0)	5 (21.7)
Initial Surgery, *n* (%)	14 (93.3)	6 (75.0)	19 (82.6)
Repeat Surgery, *n* (%)	1 (6.7)	2 (25.0)	4 (17.4)
Need for Rehabilitation	10 (66.7)	3 (37.5)	13 (56.5)
Spinal Level			
C + CTh, *n* (%)	11 + 1 (80)	4 + 0 (50)	15 + 1 (69.6)
Th + ThL, *n* (%)	3 + 0 (20)	3 + 1 (50)	6 + 1 (30.4)
L + LS, *n* (%)	0 (0)	0 (0)	0 (0)
Number of Levels of Extension			
1, *n* (%)	7 (46.7)	4 (50)	11 (47.8)
2, *n* (%)	4 (26.7)	3 (37.5)	7 (30.4)
3, *n* (N)	2 (13.3)	0 (0)	2 (8.7)
4, *n* (%)	2 (13.3)	1 (12.5)	3 (13.0)
Motor Weakness (Lovett Scale Deterioration)			
Before Surgery, *n* (%)	3 (20.0)	1 (12.5)	4 (17.4)
At Discharge, *n* (%)	8 (53.3)	2 (25.0)	10 (43.5)
Improvement, *n* (%)	2 (13.3)	1 (12.5)	3 (13.0)
Unchanged, *n* (%)	8 (53.4)	6 (75.0)	14 (60.9)
Deterioration, *n* (%)	5 (33.3)	1 (12.5)	6 (26.1)

* Hemangioblastoma WHO I, 4; Astrocytoma WHO II, III, 3; Meningioma psammomatosum WHO I, 1. Abbreviations: ID-IM, intradural intramedullary; GTR, gross total resection; STR, subtotal resection; C, cervical; Th, thoracic; L, lumbar; S, sacral.

## Appendix B

**Table A2:** Characteristics of the ID-EM group.

	NST *	Meningioma	Ependymoma	Other Tumors ^	Total	*p*-Value
*n*	24 (38.7)	17 (27.4)	11 (17.7)	10 (16.1)	62 (100)	
Female, *n* (%)	12 (50.0)	15 (88.2)	7 (63.6)	3 (30.0)	37 (59.7)	0.023
Male, *n* (%)	12 (50.0)	2 (17.8)	4 (36.4)	7 (70.0)	25 (40.3)	
Age, Mean ± SD [Range]	47.8 ± 15.51 [26–72]	64.1 ± 12.56 [32–83]	51.1 ± 16.67 [18–74]	47.7 ± 12.97 [27–65]	52.8 ± 16.20 [18–83]	××
MMS Before Surgery, Mean ± SD	1.4 ± 0.70	2.1 ± 1.26	1.3 ± 0.62	1.8 ± 0.98	1.6 ± 0.97	×
MMS at Discharge, Mean ± SD	1.3 ± 0.84	1.9 ± 1.13	1.3 ± 0.62	1.5 ± 0.81	1.5 ± 0.93	×
Length of Hospital Stay, Mean ± SD [Range]	6.5 ± 1.76 [3–21]	6.9 ± 1.76 [5–10]	7.7 ± 3.41 [5–16]	7.2 ± 1.62 [4–9]	6.95 ± 2.95 [3–21]	″
GTR, *n* (%)	22 (91.7)	10 (58.8)	9 (81.8)	8 (80.0)	49 (79.0)	0.055
STR, *n* (%)	2 (8.3)	7 (41.2)	2 (18.2)	2 (20.0)	13 (21.0)	
Initial Surgery, *n* (%)	16 (66.6)	11 (64.7)	8 (72.7)	4 (40.0)	39 (62.9)	0.215
Repeat Surgery, *n* (%)	8 (33.3)	6 (35.3)	3 (27.3)	6 (60.0)	23 (37.1)	
Need for Rehabilitation, *n* (%)	2 (8.3)	6 (35.3)	2 (18.2)	2 (20.0)	12 (19.4)	0.239
Spinal Level						
C + CTh, *n* (%)	7 + 0 (29.2)	2 + 2 (23.5)	0 (0)	0 (0)	9 + 2 (17.7)	0.069
Th + ThL, *n* (%)	8 + 0 (33.3)	12 + 0 (70.6)	1 + 1 (18.2)	3 + 2 (50.0)	24 + 3 (43.5)	0.027
L + LS, *n* (%)	7 + 2 (37.5)	0 + 1 (5.9)	9 + 0 (81.8)	5 + 0 (50.0)	21 + 3 (38.7)	0.001
Number of Levels of Extension						
1, *n* (%)	13 (54.2)	7 (41.2)	5 (45.5)	0 (0)	25 (40.3)	0.032
2, *n* (%)	8 (33.3)	6 (35.3)	4 (36.4)	9 (90)	27 (43.5)	0.015
3, *n* (%)	3 (12.5)	4 (23.5)	2 (18.2)	1 (10)	10 (16.1)	0.744
Motor Weakness (Lovett Scale Deterioration)						
Before Surgery, *n* (%)	7 (29.2)	7 (41.2)	3 (27.3)	4 (40)	21 (33.9)	0.795
At Discharge, *n* (%)	5 (20.8)	6 (35.3)	2 (18.2)	3 (30)	16 (25.8)	0.674
Improvement, *n* (%)	2 (8.3)	3 (17.6)	2 (18.2)	2 (30)	9 (14.5)	0.744
Unchanged, *n* (%)	21 (87.5)	14 (82.4)	8 (72.7)	7 (60)	50 (80.6)	0.588
Deterioration, *n* (%)	1 (4.2)	0 (0.0)	1 (9.1)	1 (10)	3 (4.8)	0.593

* NST, nerve sheath tumors: schwannoma, 21; neurofibroma, 3. ^ chordoma, cystis epidermialis, hemangioblastoma, 2; lipoma simplex, MPNST, neuroma, teratoma maturum, 1. × age difference meningioma vs. all other groups: *p* < 0.05; differences between all other groups: *p* > 0.05. ″ all differences between all groups: *p* > 0.05. ×× MMS before surgery and at discharge difference meningioma vs. NST: *p* = 0.028; differences between all other groups: *p* > 0.05. Abbreviations: ID-EM, intradural extramedullary; GTR, gross total resection; STR, subtotal resection; MPNST, malignant peripheral nerve sheath tumor; C, cervical; Th, thoracic; L, lumbar; S, sacral.

## References

[1] Louis D.N., Perry A., Reifenberger G., von Deimling A., Figarella-Branger D., Cavenee W.K., Ohgaki H., Wiestler O.D., Kleihues P., Ellison D.W. (2016). The 2016 World Health Organization Classification of Tumors of the Central Nervous System: A summary. Acta Neuropathol..

[2] Miller K.D., Ostrom Q.T., Kruchko C., Patil N., Tihan T., Cioffi G., Fuchs H.E., Waite K.A., Jemal A., Siegel R.L. (2021). Brain and other central nervous system tumor statistics, 2021. CA Cancer J. Clin..

[3] Van Goethem J.W., van den Hauwe L., Ozsarlak O., De Schepper A.M., Parizel P.M. (2004). Spinal tumors. Eur. J. Radiol..

[4] Carlos-Escalante J.A., Paz-López Á.A., Cacho-Díaz B., Pacheco-Cuellar G., Reyes-Soto G., Wegman-Ostrosky T. (2022). Primary Benign Tumors of the Spinal Canal. World Neurosurg..

[5] Chamberlain M.C., Tredway T.L. (2011). Adult primary intradural spinal cord tumors: A review. Curr. Neurol. Neurosci. Rep..

[6] Ottenhausen M., Ntoulias G., Bodhinayake I., Ruppert F.H., Schreiber S., Förschler A., Boockvar J.A., Jödicke A. (2019). Intradural spinal tumors in adults-update on management and outcome. Neurosurg. Rev..

[7] Raj V.S., Lofton L. (2013). Rehabilitation and treatment of spinal cord tumors. J. Spinal Cord. Med..

[8] Hsu S., Quattrone M., Ostrom Q., Ryken T.C., Sloan A.E., Barnholtz-Sloan J.S. (2011). Incidence patterns for primary malignant spinal cord gliomas: A Surveillance, Epidemiology, and End Results study. J. Neurosurg. Spine.

[9] Yüce İ., Kahyaoğlu O., Çavuşoğlu H.A., Ataseven M., Çavuşoğlu H., Aydın Y. (2021). Surgical treatment and outcomes of intramedullary tumors by minimally invasive approach. J. Clin. Neurosci..

[10] Svoboda N., Bradac O., de Lacy P., Benes V. (2018). Intramedullary ependymoma: Long-term outcome after surgery. Acta Neurochir..

[11] Narayan S., Rege S.V., Gupta R. (2021). Clinicopathological Study of Intradural Extramedullary Spinal Tumors and Its Correlation with Functional Outcome. Cureus.

[12] Schaller B. (2005). Spinal meningioma: Relationship between histological subtypes and surgical outcome?. J. Neurooncol..

[13] Hohenberger C., Hinterleitner J., Schmidt N.O., Doenitz C., Zeman F., Schebesch K.M. (2020). Neurological outcome after resection of spinal schwannoma. Clin. Neurol. Neurosurg..

[14] Garcés-Ambrossi G.L., McGirt M.J., Mehta V.A., Sciubba D.M., Witham T.F., Bydon A., Wolinksy J.P., Jallo G.I., Gokaslan Z.L. (2009). Factors associated with progression-free survival and long-term neurological outcome after resection of intramedullary spinal cord tumors: Analysis of 101 consecutive cases. J. Neurosurg. Spine.

[15] McCormick P.C., Torres R., Post K.D., Stein B.M. (1990). Intramedullary ependymoma of the spinal cord. J. Neurosurg..

[16] Hongo H., Takai K., Komori T., Taniguchi M. (2018). Intramedullary spinal cord ependymoma and astrocytoma: Intraoperative frozen-section diagnosis, extent of resection, and outcomes. J. Neurosurg. Spine.

[17] Brotchi J., Fischer G. (1998). Spinal cord ependymomas. Neurosurg. Focus.

[18] Raco A., Esposito V., Lenzi J., Piccirilli M., Delfini R., Cantore G. (2005). Long-term follow-up of intramedullary spinal cord tumors: A series of 202 cases. Neurosurgery.

[19] Klekamp J. (2015). Spinal ependymomas. Part 1: Intramedullary ependymomas. Neurosurg. Focus.

[20] Butenschoen V.M., Gloßner T., Hostettler I.C., Meyer B., Wostrack M. (2022). Quality of life and return to work and sports after spinal ependymoma resection. Sci. Rep..

[21] Behmanesh B., Gessler F., Won S.Y., Dubinski D., Quick-Weller J., Imoehl L., Seifert V., Marquardt G. (2020). Return to work and clinical outcome after surgical treatment and conservative management of patients with intramedullary spinal cord ependymoma. Sci. Rep..

[22] Catz A., Goldin D., Fishel B., Ronen J., Bluvshtein V., Gelernter I. (2004). Recovery of neurologic function following nontraumatic spinal cord lesions in Israel. Spine.

[23] Momin A.A., Oyem P., Patil N., Soni P., Potter T.O., Cioffi G., Waite K., Ostrom Q., Kruchko C., Barnholtz-Sloan J.S. (2022). Epidemiology of primary malignant non-osseous spinal tumors in the United States. Spine J..

[24] Abdullah K.G., Lubelski D., Miller J., Steinmetz M.P., Shin J.H., Krishnaney A., Mroz T.E., Benzel E.C. (2015). Progression free survival and functional outcome after surgical resection of intramedullary ependymomas. J. Clin. Neurosci..

[25] Klekamp J. (2015). Spinal ependymomas. Part 2: Ependymomas of the filum terminale. Neurosurg. Focus.

[26] Xia L.L., Tang J., Huang S.L. (2021). Primary intraspinal benign tumors treated surgically: An analysis from China. Br. J. Neurosurg..

[27] Jinnai T., Koyama T. (2005). Clinical characteristics of spinal nerve sheath tumors: Analysis of 149 cases. Neurosurgery.

[28] DiGiorgio A.M., Virk M.S., Mummaneni P.V. (2020). Spinal meningiomas. Handb. Clin. Neurol..

[29] Santos R.C., de Amoreira Gepp R. (2018). Benefits of spinal meningioma resection. Surg. Neurol. Int..

[30] Haegelen C., Morandi X., Riffaud L., Amlashi S.F., Leray E., Brassier G. (2005). Results of spinal meningioma surgery in patients with severe preoperative neurological deficits. Eur. Spine J..

[31] Setzer M., Vatter H., Marquardt G., Seifert V., Vrionis F.D. (2007). Management of spinal meningiomas: Surgical results and a review of the literature. Neurosurg. Focus.

[32] Sacko O., Rabarijaona M., Loiseau H. (2008). Spinal meningioma surgery after 75 years of age. Neurochirurgie.

[33] Adler S.S., Beckers D., Buck M. (2023). PNF in Practice.

[34] Krajewski S., Furtak J., Zawadka-Kunikowska M., Kachelski M., Birski M., Harat M. (2022). Comparison of the Functional State and Motor Skills of Patients after Cerebral Hemisphere, Ventricular System, and Cerebellopontine Angle Tumor Surgery. Int. J. Environ. Res. Public Health.

[35] Peus D., Newcomb N., Hofer S. (2013). Appraisal of the Karnofsky Performance Status and proposal of a simple algorithmic system for its evaluation. BMC Med. Inform. Decis. Mak..

[36] Schag C.C., Heinrich R.L., Ganz P.A. (1984). Karnofsky performance status revisited: Reliability, validity, and guidelines. J. Clin. Oncol..

[37] Patel P., Mehendiratta D., Bhambhu V., Dalvie S. (2021). Clinical outcome of intradural extramedullary IDeM spinal cord tumors: A single-center retrospective analytical study. Surg. Neurol. Int..

[38] Riad H., Knafo S., Segnarbieux F., Lonjon N. (2013). Spinal meningiomas: Surgical outcome and literature review. Neurochirurgie.

[39] Solero C.L., Fornari M., Giombini S., Lasio G., Oliveri G., Cimino C., Pluchino F. (1989). Spinal meningiomas: Review of 174 operated cases. Neurosurgery.

[40] New P.W., Marshall R., Stubblefield M.D., Scivoletto G. (2017). Rehabilitation of people with spinal cord damage due to tumor: Literature review, international survey and practical recommendations for optimizing their rehabilitation. J. Spinal Cord. Med..

[41] Pataraia A., Crevenna R. (2019). Challenges in rehabilitation of patients with nontraumatic spinal cord dysfunction due to tumors: A narrative review. Wien. Klin. Wochenschr..

[42] Fortin C.D., Voth J., Jaglal S.B., Craven B.C. (2015). Inpatient rehabilitation outcomes in patients with malignant spinal cord compression compared to other non-traumatic spinal cord injury: A population based study. J. Spinal Cord. Med..

[43] New P.W., Reeves R.K., Smith É., Eriks-Hoogland I., Gupta A., Scivoletto G., Townson A., Maurizio B., Post M.W. (2016). International Retrospective Comparison of Inpatient Rehabilitation for Patients with Spinal Cord Dysfunction: Differences According to Etiology. Arch. Phys. Med. Rehabil..

